# The Impact of *Mycobacterium avium* subsp. *paratuberculosis* on Intestinal Microbial Community Composition and Diversity in Small-Tail Han Sheep

**DOI:** 10.3390/pathogens13121118

**Published:** 2024-12-18

**Authors:** Shi-Yuan Xue, Wei Ma, Meng-Yuan Li, Wei-Kang Meng, Yu-Lin Ding, Bo Yang, Yue-Rong Lv, Rui-Bin Chen, Zhi-Hong Wu, Siqin Tunala, Rong Zhang, Li Zhao, Yong-Hong Liu

**Affiliations:** 1 College of Veterinary Medicine, Inner Mongolia Agricultural University, Hohhot 010010, China; xsy55145600@163.com (S.-Y.X.); 15624780206@163.com (W.M.); 13213508200@163.com (M.-Y.L.); 15848155638@163.com (W.-K.M.); dingyulin2001@126.com (Y.-L.D.); lyr765619420@163.com (Y.-R.L.); 2 Key Laboratory of Clinical Diagnosis and Treatment Technology in Animal Disease, Ministry of Agriculture and Rural Affairs, Hohhot 010018, China; 3 Animal Disease Control Center of Ordos, Ordos 017000, China; 15886840959@126.com; 4 Otok Banner Animal Disease Prevention and Control Center, Ordos 017000, China; cyb_0921@163.com (R.-B.C.); nmhfei@126.com (S.T.); zr_129@163.com (R.Z.); 5 Agriculture and Animal Husbandry Technology Popularization Center of Inner Mongolia Autonomous Region, Hohhot 010010, China; wzh8410051@163.com

**Keywords:** *Mycobacterium avium* subsp. *paratuberculosis*, small-tail Han sheep, microbial population, high-throughput sequencing, China

## Abstract

Paratuberculosis (PTB), primarily caused by *Mycobacterium avium* subsp. *paratuberculosis* (MAP), is a chronic infection that affects ruminants and is difficult to prevent, diagnose, and treat. Investigating how MAP infections affect the gut microbiota in sheep can aid in the prevention and treatment of ovine PTB. This study examined fecal samples from eight small-tail Han sheep (STHS) at various stages of infection and from three different field areas. All samples underwent DNA extraction and 16S rRNA sequencing. Among all samples, the phyla p. Firmicutes and p. Bacteroidota exhibited the highest relative abundance. The dominant genera in groups M1–M6 were UCG-005, *Christensenellaceae*_R-7_group, *Rikenellaceae*_RC9_gut_group, *Akkermansia*, UCG-005, and *Bacteroides*, whereas those in groups A–C were *Christensenellaceae*_R-7_group, *Escherichia–Shigella*, and *Acinetobacter*, respectively. The microbial community structure varied significantly among groups M1–M6. Specifically, 56 microbiota consortia with different taxonomic levels, including the order Clostridiales, were significantly enriched in groups M1–M6, whereas 96 microbiota consortia at different taxonomic levels, including the family *Oscillospiraceae*, were significantly enriched in groups A–C. To the best of our knowledge, this is the first study to report that MAP infection alters the intestinal microbiota of STHS. Changes in p. Firmicutes abundance can serve as a potential biomarker to distinguish MAP infection and determine the infection stage for its early diagnosis. Our study provides a theoretical basis for the treatment of PTB by regulating the intestinal microbiota, including p. Firmicutes.

## 1. Introduction

Paratuberculosis (PTB), also known as Johne’s disease [1], is caused by the pathogen *Mycobacterium avium* subsp. *paratuberculosis* (MAP) [2]. MAP is primarily transmitted via the fecal–oral route [3]. It can persist in the macrophages of the small intestinal mucosa [4] and cause granulomatosis in the intestinal and mesenteric lymph nodes [5]. Ruminants with PTB clinically present with untreatable diarrhea, which eventually leads to death [6]. Additionally, MAP has been linked to Crohn’s disease (CD) [7], type 1 diabetes (T1D), and certain autoimmune diseases [2]. There is increasing evidence that MAP is a zoonotic pathogen [8].

The intestinal microecosystem comprises intestinal microbiota and its surrounding environment and helps maintain internal environmental homeostasis in humans and animals [9]. The intestinal microbiota coexists with the host, facilitating the absorption and digestion of nutrients, maintaining the integrity of the host’s immune system, and preventing the invasion of pathogenic microorganisms [10]. Normally, the intestinal microbiota maintains a state of dynamic equilibrium; however, illness can disrupt this equilibrium, leading to a disturbance in the microbiota [11]. For example, dysbiosis is prevalent in patients with CD [12]. According to a previous study, the abundance of the phylum (p.) Proteobacteria increased while that of *p*. Firmicutes decreased in the colon biopsy specimens of patients with HIV-1 [13]. Furthermore, chickens infected with avian influenza A virus H9N2 showed an increased abundance of p. Proteobacteria [14]; Marek’s virus also affects the diversity of the intestinal microbiota of chickens [15]. Additionally, Porcine epidemic diarrhea virus [16] and *Salmonella* [17] can cause changes in the intestinal microbiota of swine. It has also been reported that *Toxoplasma* [18] and *Helicospira polymorpha* [19] alter the composition of the intestinal microbiota in mice. Studies have suggested that ruminant infection with MAP also causes MAP with intestinal dysbiosis [20], and these infections can significantly reduce microbial richness [21]. Moreover, the fecal bacterial composition of MAP-positive cows varies significantly compared to that of MAP-negative cows [22]. Another study indicated that the relative abundances of the genera (g.) *Akkermansia*, g. *Faecalibacterium*, g. *Planococcaceae* (p. Firmicutes), and g. CF231 (p. Bacteroidetes) increased significantly in calves after infection with MAP [23].

Small-tail Han sheep (STHS) are widely bred regional sheep in China [24], characterized by year-round estrus and hyperprolificacy [25]. In the cecum and rectum of STHS, p. Firmicutes and p. Bacteroidetes are the dominant phyla whereas g. *Bacteroidetes*, g. *Ruminococcus*, g. *Lactobacillus*, g. *Flavonifractor*, and g. *Clostridium* are the dominant genera [26]. To the best of our knowledge, there are currently no reports on changes in sheep gut microbiota following infection with MAP. In this study, high-throughput sequencing technology was used to rapidly and efficiently [27] analyze the structural changes in the STHS intestinal microbial community after artificial inoculation and natural infection with MAP. The results may provide a theoretical basis for the prevention and treatment of PTB by regulating the intestinal microbiota.

## 2. Materials and Methods

### 2.1. Sample Collection

The first experiment involved samples obtained from the established animal infection model of ovine PTB in our laboratory (results to be published). Eight 3-month-old lambs (STHS, from a sheep farm in Hohhot) were challenged with sheep-derived Type II MAP. Group A consisted of sheep No. 1–4, with an inoculum dose of approximately 9.2 × 10^8^ CFU live bacteria per sheep. Group B consisted of sheep No. 5–8, with an inoculum dose of approximately 2.57 × 10^9^ CFU live bacteria per sheep. The post-inoculation period refers to the time elapsed since the initial inoculation of MAP. Eight sheep were sampled at six distinct time points (prior to inoculation and at 3, 60, 90, 120, and 150 days post-inoculation). A total of 48 fecal samples were collected from the rectum and labeled as groups M1–M6, corresponding to the six aforementioned sampling time points (Table 1).

The second experiment involved collecting fecal samples from STHS at three sampling sites (all livestock farms): group A, group B, and group C. These sampling sites were located in Western Inner Mongolia (group A), Central Inner Mongolia (group B), and Eastern Inner Mongolia (group C). Multiple 1.5-year-old STHS were randomly selected at each sampling site for fecal sample collection.

### 2.2. DNA Extraction and Nested PCR

Fecal samples collected from the field were subjected to DNA extraction within biosafety cabinets, strictly following the protocols outlined by the manufacturer of the E.Z.N.A Stool DNA Kit (Omega BioTek Inc., Norcross, GA, USA). Three rounds of nested PCR targeting the *IS900* gene of MAP [28] were performed using Premix Taq™ (TaKaRa Taq™ Version 2.0 plus dye) (TaKaRa, Beijing, China) to determine the presence of MAP in the fecal samples. Based on the nested PCR results from the second experiment, 24 samples were selected for high-throughput sequencing (Table 1).

### 2.3. High-Throughput Sequencing

The DNA from a portion of each sample was used as a template to amplify the 16S rDNA V3–V4 hypervariable region using specific primers with a barcode and the TransStart^®^ FastPfu DNA Polymerase (TransStart, Beijing, China, Code No. AP221-02). The universal primers were 338F (5′-ACT CCT ACG GGA GGC AGC A-3′) and 806R (5′-GGA CTA CHV GGG TWT CTA AT-3′). The volume of the PCR was 25 μL, with an annealing temperature of 55 °C. The AXYPREP DNA Gel Extraction Kit (AXYGEN, Suzhou, China, Code No. AP-GX-50G) was used to purify the PCR products. First, the PCR products were quantified via 2% agarose gel electrophoresis. Next, they were detected and quantified using the QuantiFluor™-ST Blue fluorescence quantification system (Promega, Madison, WI, USA). The corresponding proportions of PCR products were mixed based on the sequencing volume requirements for each sample. The VAHTS^®^ ssDNA Library Prep Kit (Illumina, San Diago, CA, USA, Code No. ND6201) was then used to construct the Illumina PE250 library. Finally, 16S rRNA sequencing was performed using the Illumina Novaseq 6000 platform (San Diego, CA, USA). Sequencing was conducted in collaboration with Origin-gene Biology Co., Ltd., (Shanghai, China).

### 2.4. Data Analysis

The paired-end reads obtained via Illumina PE250 sequencing were first assembled based on overlap relationships, quality control, and sequence quality filtering. Next, operational taxonomic unit (OTU) clustering, species classification, and diversity index analyses were performed. Sequencing depth was also assessed, statistical analysis of community structure at each taxonomic level was conducted, and a series of visual analyses were performed. Sequencing analysis was performed using the UPARSE software package [29] (V7.0.1090, http://drive5.com/uparse/, accessed on 25 April 2024). Sequences were assigned to the same OTUs at 97% similarity, and taxonomic analysis of OTU representative sequences from the phylum level to the species level was performed using the Qiime platform (V1.9.0, http://qiime.org/scripts/assign_taxonomy.html, accessed on 25 April 2024). The RDP classifier [30] (V2.2, http://sourceforge.net/projects/rdp-classifier/, accessed on 25 April 2024) and Bayesian algorithm were used to perform a taxonomic analysis of OTU representative sequences from the phylum level to the species level. Additionally, databases such as Silva [31] (Release132, http://www.arb-silva.de, accessed on 25 April 2024), RDP [32] (Release 11.5, http://rdp.cme.msu.edu/, accessed on 25 April 2024), Greengenes [33] (Release 13.8, http://greengenes.secondgenome.com/, accessed on 25 April 2024), Unite [34] (Release 7.1, http://unite.ut.ee/index.php, accessed on 25 April 2024), FGR [35], and RDP (Release 7.3, http://fungene.cme.msu.edu/, accessed on 25 April 2024) were used to align sequences with those from GenBank. Alpha diversity analysis of individual samples was performed on the mothur website [36] (V1.30.1, http://www.mothur.org/wiki/Schloss_SOP#Alpha_diversity, accessed on 25 April 2024). Metrics such as the number of unique OTUs per sample, Chao1 index of community richness (http://www.mothur.org/wiki/Chao, accessed on 25 April 2024), Shannon index of community diversity (http://www.mothur.org/wiki/Shannon, accessed on 25 April 2024), Simpson index (http://www.mothur.org/wiki/Simpson, accessed on 25 April 2024), sequencing depth index, Good’s coverage (http://www.mothur.org/wiki/Coverage, accessed on 25 April 2024), and community composition were analyzed using statistical methods, and community structures at different taxonomic levels were visualized using bar graphs [37]. Beta diversity analysis of the samples was conducted based on the UniFrac principal coordinate analysis (PCoA) [38]; potential principal components affecting sample community composition were identified at the evolutionary level based on the evolutionary distance. Linear discriminant analysis (LDA) effect size (LEfSe) analysis was used to detect high-dimensional biological identifiers, determine genomic features [39], and perform LDA for different sample groups based on taxonomic composition analysis [40] (http://huttenhower.sph.harvard.edu/galaxy/root?tool_id=lefse_upload, accessed on 25 April 2024). This was performed to determine the community or species with significant differential effects on sample grouping.

## 3. Results

### 3.1. Sequencing Data Statistics

After the sequencing results of the first experiment (48 samples) were corrected and the chimeras were removed, between 43,005 and 144,951 optimized sequences were obtained. The optimized data for bases per sample ranged from 17,709,323 to 59,411,061 bp, with an average length of 412.01 bp, and 99.46% of the sequences were assigned lengths between 401 and 440 bp. For the second experiment (24 samples), after the correction of the results and the removal of the chimeras, between 31,949 and 393,633 optimized sequences were obtained. The optimized data for bases per sample ranged from 13,149,785 to 166,841,328 bp, with an average length of 415.57 bp, and 99.82% of the sequences were assigned lengths between 401 and 440 bp (Table S1). Across all 72 samples, at a 97% similarity threshold, 3,482,765 high-quality sequences were generated through OTU selection and taxonomic assignments, resulting in clean reads and 84,555 effective OTUs. In the first experiment, 93.13% of the OTU sequences were assigned to the genus level. In the second experiment, groups A, B, and C had 91.86%, 94.80%, and 93.23% of the OTU sequences assigned to the genus level, respectively.

### 3.2. Alpha Diversity Analysis

Alpha diversity, including the Shannon, Simpson, Chao, ACE, and coverage indices, was calculated based on a 97% similarity threshold. In the first experiment, sample 6-5 had the largest Shannon index and the smallest Simpson index, whereas sample 1-5 had the largest ACE and Chao indices. In the second experiment, sample B6 had the largest Shannon index and the smallest Simpson index, whereas sample C7 had the largest ACE and Chao indices. In both experiments, it was observed that the larger the Shannon index, the smaller the Simpson index. Furthermore, the Chao and ACE indices were ranked consistently, from large to small, and Good’s coverage in both experiments exceeded 98.69% (Table S2).

### 3.3. Microbial Population

The 48 samples from the first experiment were annotated to 18 phyla, 13 of which appeared in all samples. The 24 samples from the second experiment were annotated to 25 phyla, 22 of which appeared in all samples. Across all samples, p. Firmicutes exhibited the highest relative abundance, followed by p. Bacteroidota (Table 2).

The samples from the first experiment were annotated to 224 genera, 196 of which appeared in all samples. The dominant genera in groups M1–M6 were g. UCG-005, g. *Christensenellaceae*_R-7_group, g. *Rikenellaceae*_RC9_gut_group, g. *Akkermansia*, g. UCG-005, and g. *Bacteroides*, respectively. The samples from the second experiment were annotated to 698 genera, 365 of which appeared in all samples. The dominant genera in groups A–C were g. Christensenellaceae_R-7_group, g. *Escherichia-Shigella*, and g. *Acinetobacter*, respectively (Table 3).

### 3.4. Beta Diversity Analysis

**Figure 1:**
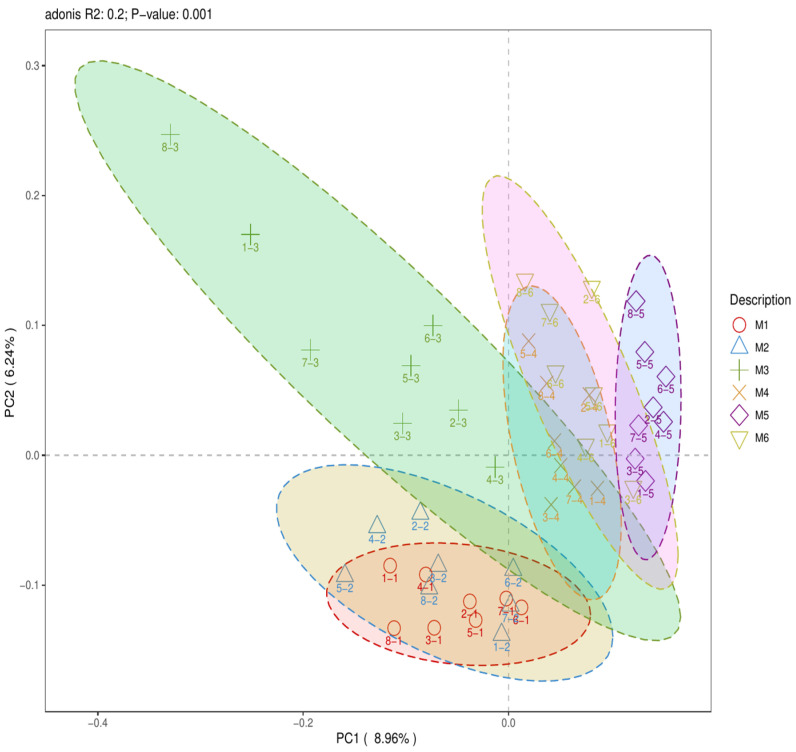
Two-dimensional PCoA plot of groups M1–M6.

**Figure 2:**
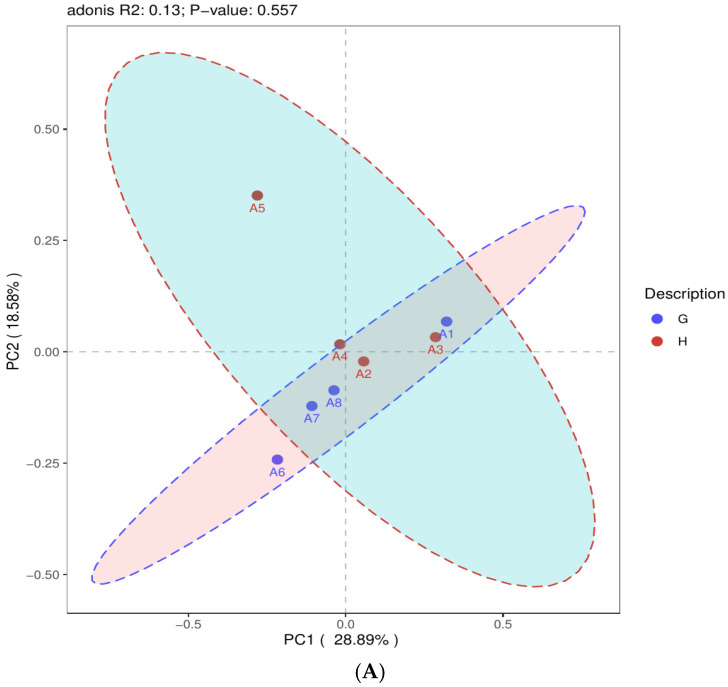

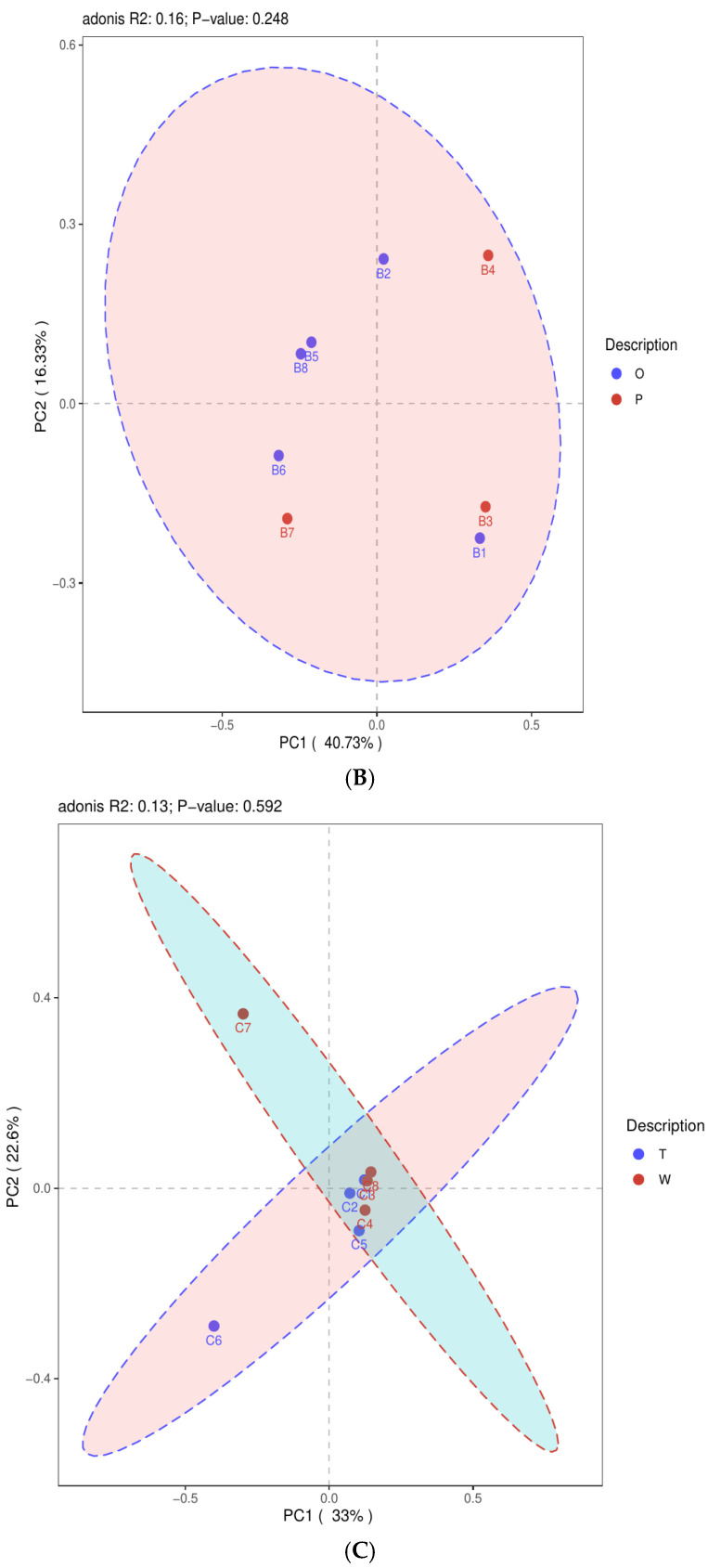
(**A**) Two-dimensional PCoA plot of group A. Blue solid circle (●) (G): MAP-negative samples. Red solid circles (●) (H): MAP-positive samples. (**B**) Two-dimensional PCoA plot of group B. Red solid circle (●) (P): MAP-negative samples. Blue solid circle (●) (O): MAP-positive samples. (**C**) Two-dimensional PCoA plot of group C. Red solid circles (●) (W): MAP-negative samples. Blue solid circles (●) (T): MAP-positive samples.

Using a 95% confidence interval, PCoA was performed on two experimental datasets according to different MAP detection results from samples at the same location (groups A–C) and different MAP infection stages (groups M1–M6) with different grouping clustering patterns. PCoA of groups M1–M6 showed an R^2^ value of 0.2 and a *p*-value of 0.001 (*p* < 0.001) (Figure 1). For the second experiment (with groups A–C), the number of positive and negative samples detected by nested PCR in each group was ≥3. PCoA of positive and negative samples in groups A–C revealed R^2^ values of 0.13, 0.16, and 0.13 and *p*-values of 0.557 (*p* > 0.05), 0.248 (*p* > 0.05), and 0.592 (*p* > 0.05), respectively (Figure 2A–C).

### 3.5. LEfSe Analysis

**Figure 3:**
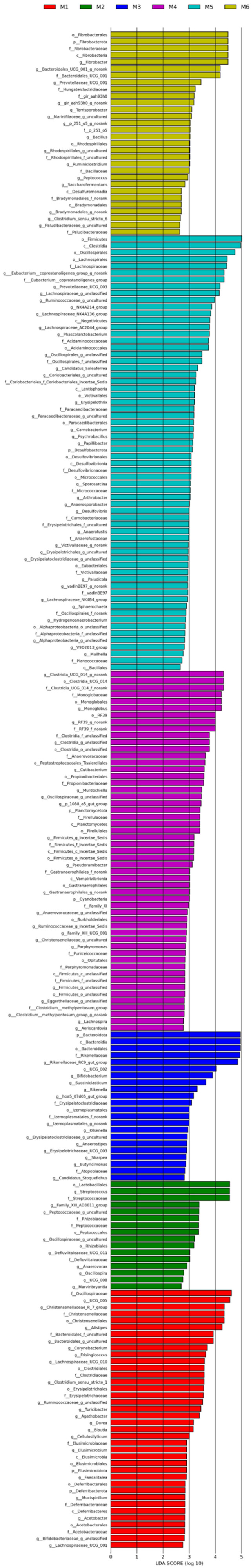
LDA analysis of groups M1–M6.

In the first experiment, group M1 had 38 microbial communities at different taxonomic levels, including the family (f.) *Oscillospiraceae* (*p* < 0.05), that were significantly enriched. In group M2, 16 microbial communities at different taxonomic levels, including f. *Streptococcaceae* (*p* < 0.05), were significantly enriched. In group M3, 22 microbial communities at different taxonomic levels, including g. *Olsenella* (*p* < 0.05), were significantly enriched. In group M4, 53 microbial communities at different taxonomic levels, including the order (o.) Clostridia_o_unclassified (*p* < 0.05), were significantly enriched. In group M5, 64 microbial communities at different taxonomic levels, including g. *Lachnospiraceae*_NK4B4_group (*p* < 0.05), were significantly enriched. Finally, in group M6, 30 microbial communities at different taxonomic levels, including f. *Rhodospirillales*_f_uncultured (*p* < 0.05), were significantly enriched (Figure 3).

**Figure 4:**
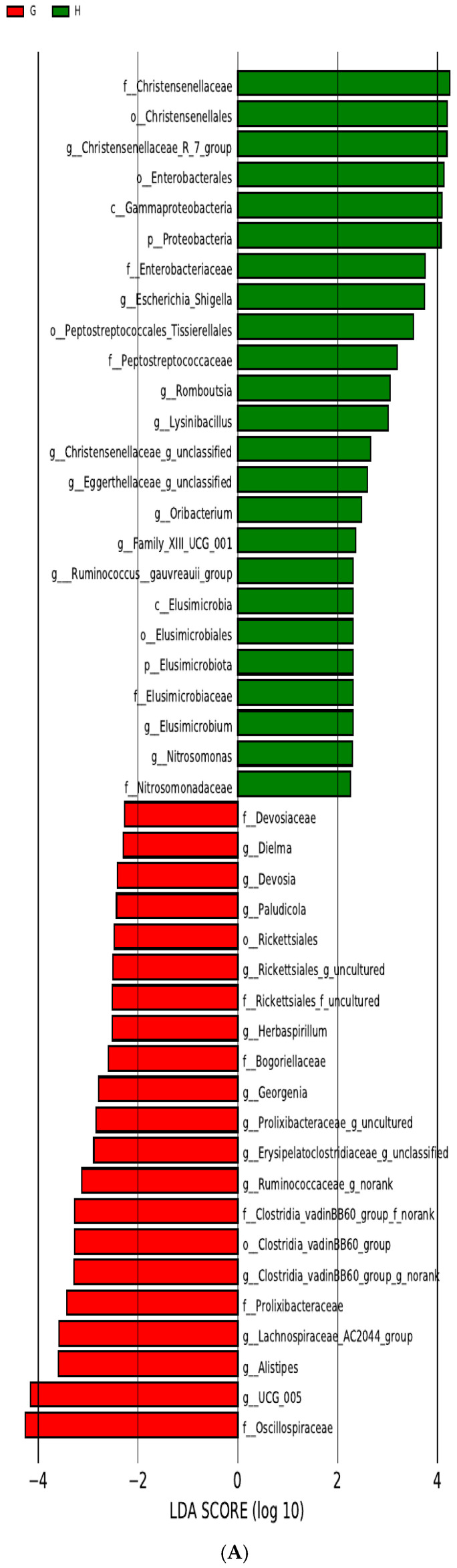

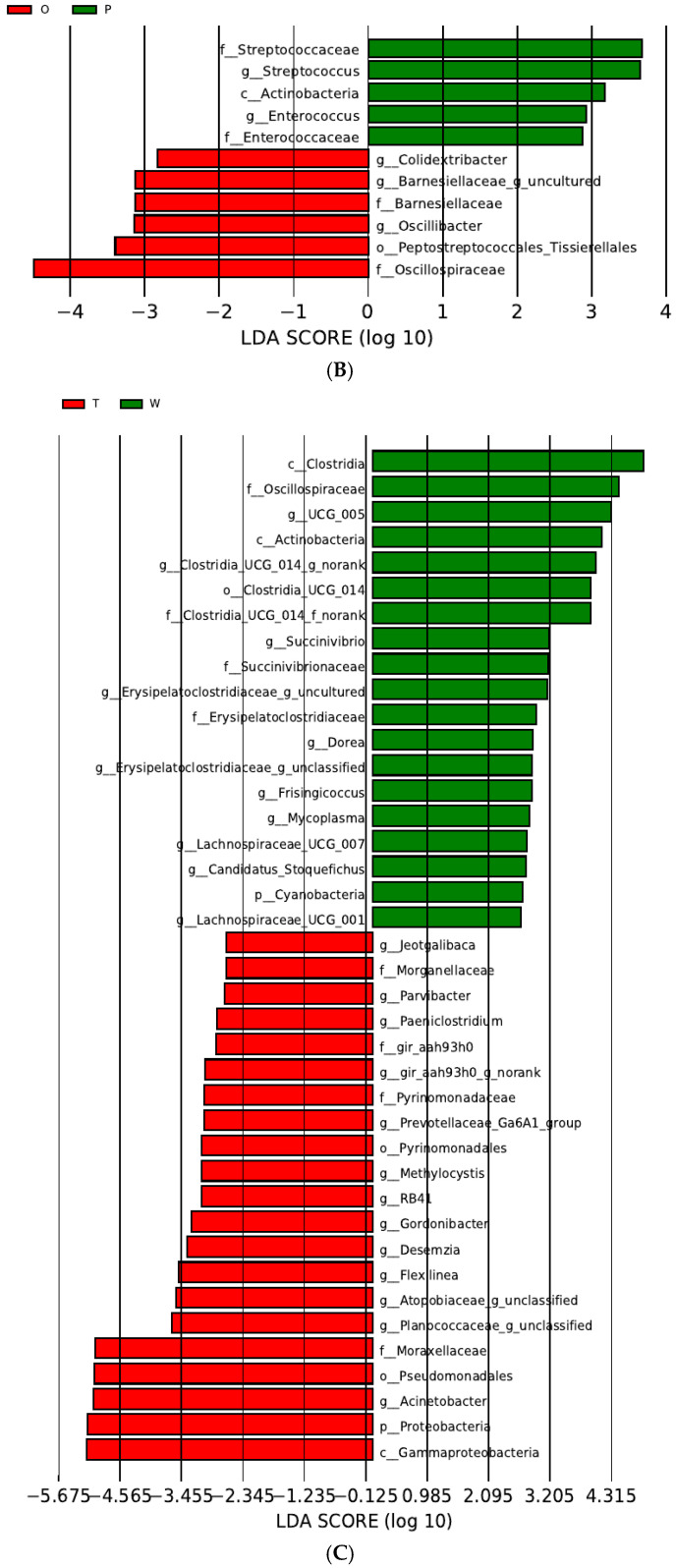
(**A**). LDA analysis of group A. Red rectangle (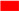) (G): MAP-negative samples. Green rectangle (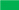) (H): MAP-positive samples. (**B**). LDA analysis of group B. Green rectangle (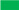) (P): MAP-negative samples. Red rectangle (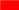) (O): MAP-positive samples. (**C**). LDA analysis of group C. Green rectangle (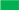) (W): MAP-negative samples. Red rectangle (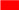) (T): MAP-positive samples.

In the second experiment, 21 microbial communities at different taxonomic levels, including the order (*o*.) Clostridia_vadinBB60_group (*p* < 0.05), were significantly enriched among the MAP-negative samples of group A. Among the MAP-positive samples of group A, 24 microbial communities at different taxonomic levels, including the phylum (*p*.) Proteobacteria (*p* < 0.05), were significantly enriched (Figure 4A). Among the MAP-negative samples of group B, five microbial communities at different taxonomic levels, including the class (*c.*) Actinobacteria (*p* < 0.05), were significantly enriched. Among the MAP-positive samples of group B, six microbial communities at different taxonomic levels, including o. Peptostreptococcales_Tissierellales (*p* < 0.05), were significantly enriched (Figure 4B). Among the MAP-negative samples of group C, 19 microbial communities at different taxonomic levels, including p. Cyanobacteria (*p* < 0.05), were significantly enriched. Lastly, among the MAP-positive samples of group C, 21 microbial communities at different taxonomic levels, including p. Proteobacteria (*p* < 0.05), were significantly enriched (Figure 4C).

## 4. Discussion

In 1895, Jöhne and Frothingham first discovered the presence of an acid-fast bacillus in the thickened intestinal mucosa of cattle with chronic diarrhea. This type of bacillus was difficult to distinguish from a tubercle bacillus under the microscope. In 1906, Bang identified this bacterium as Jöhne’s bacillus [41], now known as MAP. By 1920, MAP was confirmed to be widely distributed in ruminants worldwide [42]. PTB caused by MAP can lead to economic losses in many countries [43]. In China, bovine PTB was first reported in 1953 [44], and ovine PTB was first reported in 1971 [45]. To date, PTB in ruminants has been reported in many Chinese provinces [28,43,45,46,47,48,49,50]. Currently, PTB is recognized by the World Organization for Animal Health as a major global issue in the field of animal health [51]. It is considered a type of “neglected disease” [52], with no country claiming to be free from MAP [43]. However, the underreporting and underestimation of PTB prevalence are common in many countries due to the lack of formal control plans [53]. Efficient control of PTB requires a deeper understanding of the host–MAP interactions [54].

Herein, high-throughput sequencing of the 16S rRNA V3–V4 region revealed that a larger Shannon index value for a sample corresponded to a smaller Simpson index value, indicating higher microbial community diversity. Meanwhile, a larger ACE index value indicated a higher total number of species [27,55]. The Shannon, Simpson, and ACE indices for each sample in this study fell within the upper and lower limits of statistical significance, and changes in these indices aligned with the changes in community diversity and total species, as observed in the sequencing analysis.

In this study, the sequencing depth index, or Good’s coverage, of the 72 samples exceeded 98.69%, indicating that the sequencing depth was sufficient to show the microbial diversity of the samples and reflect the actual composition of the microorganisms. In addition, the microbial richness of MAP-positive and MAP-negative samples from groups A–C, as well as samples from groups M1–M6, did not show significant changes. This finding is inconsistent with the significant decrease in microbial richness in MAP-positive samples previously reported in Korea [21].

In the two experiments conducted in this study, p. Firmicutes showed the highest relative abundance, followed by p. Bacteroidota. This finding aligns with the higher relative abundance of p. Firmicutes and p. Bacteroidetes in the cecum and rectum samples of healthy STHS, as reported in a previous study [26]. At the genus level, in the first experiment sample, only groups M1 and M5 had the same genus (i.e., g. UCG-005) with the highest relative abundance; g. UCG-005 was also among the top five genera with respect to the relative abundance in groups M2–M4, M6, and A–C. Furthermore, groups A and M2 had the same genus with the highest relative abundance. The genus with the highest relative abundance in groups M1–M5 and A belonged to p. Firmicutes, whereas the genus with the highest relative abundance in groups B and C belonged to p. Proteobacteria. In addition, the genus with the highest relative abundance in group M6 belonged to p. Bacteroidota. In this study, except for group M6, the genus with the highest relative abundance in the other groups did not correspond to the genus with a higher relative abundance in the cecum and rectum of healthy STHS, i.e., g. *Bacteroides* [26]. Additionally, the influence of environmental factors on intestinal microbiota composition is critical [56]. The second experiment included three groups of samples collected from Western, Central, and Eastern Inner Mongolia, and the stage of MAP infection in STHS at the time of sampling was uncertain. Whether the observed changes in the relative abundance of microbiota in this study represent changes in STHS after MAP infection remains to be determined.

PCoA of groups M1–M6 in the first experiment resulted in an R^2^ value of 0.2 (*p*-value of 0.001), indicating that there were significant differences in microbial community structure and that extended exposure to MAP had a significant impact on the host’s gut microbial community structure. This finding is consistent with that reported by Korean scholars, who observed that cattle infection caused significant changes in gut microbial community structure after MAP infection [21]. The PCoA for the second experiment resulted in R^2^ values that were all less than 0.2, with *p*-values greater than 0.05. These results showed that there were no statistically significant differences in the microbial community structure between the MAP-positive and MAP-negative samples within groups A–C. This finding aligns with that reported by Canadian scholars, who noted no significant changes in gut microbial community structure after dairy cattle infection with MAP [23]. Of course, this could also be influenced by various factors, such as animal variety, sample size, and the stage of MAP infection in STHS.

LEfSe analysis of the samples in the first experiment showed that 38 different microbial taxonomic groups were significantly enriched in group M1. Of these, 55.26% (21/38) of the bacterial communities belonged to p. Firmicutes. In particular, 16 bacterial groups at pre-inoculation (o. Clostridiales, o. Acetobacterales, f. *Erysipelotrichaceae*, f. *Acetobacteraceae*, f. *Clostridiaceae*, g. *Acetobacter*, g. *Christensenellaceae*_R_7_group, g. *Alistipes*, g. *Lachnospiraceae*_UCG_010, g. *Bifidobacteriaceae*_g_unclassified, g. *Blautia*, g. *Turicibacter*, g. *Cellulosilyticum*, g. *Dorea*, g. *Clostridium*_sensu_stricto, and g. *Frisingicoccus* [*p* < 0.001]) were significantly more enriched than those at the other infection stages. Of these, 68.75% (11/16) of the bacterial communities belonged to p. Firmicutes. Meanwhile, 16 microbial communities with different taxonomic levels were significantly enriched in group M2, with 87.50% (14/16) of them belonging to p. Firmicutes. In particular, the relative abundance of the three bacterial groups (o. Peptococcales, f. *Peptococcaceae*, and f. *Peptococcaceae*_g_uncultured [*p* < 0.001]) at 3 days post-inoculation was significantly more enriched than that at other stages, with 66.67% (2/3) of the bacterial groups belonging to p. Firmicutes. In group M3, 22 microbial communities with different taxonomic levels were significantly enriched, with 63.64% (14/22) of them belonging to p. Firmicutes. In particular, at 60 days post-inoculation, seven bacterial groups (f. *Rikenellaceae*, g. *Olsenella*, g. *Erysipelotrichaceae*_UCG_003, g. *Bifidobacterium*, g. *Anaerostipes*, g. *Rikenellaceae*_RC9_gut_group, and g. Sharpea [*p* < 0.001]) were more enriched than those at other stages, with 42.86% (3/7) belonging to p. Firmicutes. In group M4, 53 microbial communities of different taxonomic levels, were significantly enriched, with 66.03% (35/53) belonging to p. Firmicutes. In particular, the relative abundance of four bacterial groups (f. *Clostridium*_methylpentosum_group, g. *Ruminococcaceae*_g_Incertae_Sedis, g. *Clostridium*_methylpentosum_group_g_norank, and g. Family_XIII_UCG_001 [*p* < 0.001]) at 90 days post-inoculation was significantly more enriched than that at other stages; all four groups belonged to p. Firmicutes. In group M5, 64 microbial communities with different taxonomic levels were significantly enriched, with 61.0% (39/64) belonging to p. Firmicutes. In particular, the relative abundance of 18 bacterial groups (p. Firmicutes, o. Eubacteriales, f. *Oscillospirales*, f. *Planococcaceae*, f. *Oscillospirales*_f_unclassified, f. *Anaerofustaceae*, g. *Lachnospiraceae*_NK4B4_group, g. *Lachnospiraceae*_AC2044_group, g. *Carnobacterium*, g. *Oscillospirales*_g_unclassified, g. *Psychrobacillus*, g. *Sporosarcina*, g. *Anaerofustis*, g. *Erysipelatoclostridiaceae*_g_unclassified, g. *Victivallaceae*_g_norank, g. *Ruminococcaceae*_g_uncultured, g. *Anaerosporobacter*, and g. *Papillibacter* [*p* < 0.001]) at 120 days post-inoculation were significantly more enriched than those at the other stages, with 94.44% (17/18) belonging to p. Firmicutes. In group M6, 30 microbial communities of different taxonomic levels were significantly enriched, with 26.67% (8/30) belonging to p. Firmicutes, 16.67% (5/30) belonging to p. Fibrobacterota, and 33.33% (10/30) belonging to p. Bacteroidota. In particular, at 150 days post-inoculation, the bacterial groups p. Fibrobacterota, c. Fibrobacteria, o. Fibrobacterales, f. *Hungateiclostridiaceae*, f. *Bacteroidales*_UCG_001, f. *Fibrobacteraceae*, g. *Fibrobacter*, and g. *Bacteroidales*_UCG_001_g_norank [*p* < 0.001] had a significantly higher relative abundance than those at the other stages. Of these, only f. *Hungateiclostridiaceae* belonged to p. Firmicutes, and 62.5% (5/8) of the bacterial groups belonged to p. Fibrobacterota.

In summary, this is the first study to identify the gut microbiota with potentially significant biomarkers at different stages of exposure to MAP in STHS. Most of the microbiota significantly enriched in groups M1–M5 belonged to p. Firmicutes; however, in the final stage, (group M6, 150 days post-inoculation), most of the significantly enriched microbiota belonged to p. Fibrobacterota (33.33%) rather than p. Firmicutes (26.67%). Due to the small sample size, it remains unclear which specific microbiota or combinations of microbiota serve as important biomarkers for identifying the different stages of MAP infection. These possibilities require confirmation through studies with a larger sample size.

LEfSe analysis of samples in the second experiment revealed that 45 microbial taxa at different taxonomic levels in group A were significantly different between the infected and uninfected MAP samples; 24 of these taxa belonged to p. Firmicutes, 13 to p. Proteobacteria, 5 to p. Elusimicrobiota, and 3 to p. Actinobacteriota. A total of 11 different taxonomic microbiota in group B were significantly different between the MAP-infected and uninfected samples; 8 of these differential taxa belonged to p. Firmicutes, 2 to p. Bacteroidota, and only 1 to p. Actinobacteriota (c. Actinobacteria). A total of 40 microbial taxa at different taxonomic levels in group C were significantly different between the MAP-infected and uninfected samples; 19 of these differential taxa belonged to p. Firmicutes, 9 to p. Proteobacteria, 4 to p. Actinobacteriota, 3 to p. Acidobacteriota, 3 to p. Bacteroidota, 1 to p. Chloroflexi (g. *Flexilinea*), and 1 to p. Cyanobacteria. The results of these three groups differed from those reported by Canadian scholars [23], who identified g. *Akkermansia*, g. *Faecalibactrium*, unclassified Planococcaceae, and g. CF231 as significant factors when distinguishing between MAP-positive and MAP-negative calves. These findings are also inconsistent with the results that *C. difficile* is significantly different between MAP-positive and MAP-negative cattle, as reported by Korean scholars [21]. Such discrepancies are likely due to differences in animal breeds, geographic regions, and stages of MAP infection. The findings of this study may enhance the ability to distinguish between MAP-infected and uninfected samples, potentially serving as promising biomarkers for future use.

The LEfSe analysis of the two experiments showed that MAP infection caused changes in the intestinal microbiota. Alterations in the gut microbiota may result in intestinal dysbiosis [57]. Intestinal dysbiosis is closely associated with gastrointestinal dysfunction [58], and animals infected with MAP develop diarrhea and enteritis [59]. The changes in the intestinal microbiota found in this study may be the underlying cause of the clinical manifestations in animals suffering from PTB. Additionally, in the two experiments of this study, most of the differential microbiota analyzed using LEfSe belonged to p. Firmicutes. p. Firmicutes is one of the most dominant microbial communities in the sheep gut [60,61]. This phylum contains many genes responsible for the fermentation of dietary fiber and interactions with intestinal mucosa that contribute to body homeostasis [62]. Changes in the relative abundance of p. Firmicutes may cause some inflammatory diseases [63,64]. For instance, p450, which is present in some species of p. Firmicutes [65], participates in secondary metabolic pathways [66]. p450 also has stereospecific and region-specific oxidative abilities. Many p450s are part of secondary metabolite biosynthetic gene clusters (smBGCs) and play a role in the secondary metabolite production of p. Firmicutes species [63]. The production of secondary metabolites has a significant impact on host health [67,68]. Moreover, recent studies have found that the abundance of p. Firmicutes decreases in cases of inflammatory bowel disease (IBD) [69]. To summarize, based on the current sample size and sample environment, this study found that MAP significantly affects the structure and abundance of intestinal microbiota in STHS and that these altered microbiota serve as potential biomarkers for MAP infection. In particular, p. Firmicutes may play an important role in the development of PTB and warrant further exploration of its biomarker potential in future studies. Improving the intestinal microenvironment through probiotics or microbiota transplants could help restore homeostasis and simultaneously treat or improve disease outcomes [70]. For example, fecal microbiota transplantation (FMT) is commonly used to treat gastrointestinal diseases caused by pathogenic microorganisms [71]. FMT was initially applied for *Clostridium difficile* (CDI) infection and was later considered a potential treatment for (IBD) [72], including its use in ulcerative colitis (UC), a type of IBD [73]. The intestinal microbiota plays a crucial role in the treatment of IBD [64,74], and PTB is also categorized as a type of IBD [75]. Regulating p. Firmicutes and other microbial communities may become a significant focus of future research aimed at preventing and treating PTB.

## 5. Conclusions

MAP infection was studied in 72 fecal samples of STHS from Inner Mongolia Province, China. This study revealed that MAP infection causes changes in the intestinal microbiota of STHS, particularly in those with prolonged exposure to MAP. The most significant changes in p. Firmicutes can serve as biomarkers for distinguishing MAP infection and for the early diagnosis of MAP infection, facilitating timely diagnosis and enabling early intervention to minimize harm. Regulating the intestinal microbiota, such as p. Firmicutes, could be a promising new direction for the prevention and treatment of PTB in the future.

## Figures and Tables

**Table 1:** Samples from two experiments and the MAP detection information of the second experiment.

Group	Sheep 1	Sheep 2	Sheep 3	Sheep 4	Sheep 5	Sheep 6	Sheep 7	Sheep 8
	Sheep in PTB Animal Model							
M1	1-1	2-1	3-1	4-1	5-1	6-1	7-1	8-1
M2	1-2	2-2	3-2	4-2	5-2	6-2	7-2	8-2
M3	1-3	2-3	3-3	4-3	5-3	6-3	7-3	8-3
M4	1-4	2-4	3-4	4-4	5-4	6-4	7-4	8-4
M5	1-5	2-5	3-5	4-5	5-5	6-5	7-5	8-5
M6	1-6	2-6	3-6	4-6	5-6	6-6	7-6	8-6
	Sheep exposed to MAP in the field							
A	A1	A2 *	A3 *	A4 *	A5 *	A6	A7	A8
B	B1 *	B2 *	B3	B4	B5 *	B6 *	B7	B8 *
C	C1 *	C2 *	C3	C4	C5 *	C6 *	C7	C8

Note: M1 represents the pre-inoculation group, whereas M2–M6 represent the post-inoculation groups at 3, 60, 90, 120, and 150 days, respectively. In the sample identifiers No.1–No.2, “No.1” refers to the sheep number and “No.2” refers to the M group number. In identifiers containing a letter followed by a number, the letter represents the field group number, and the number represents the sheep number. * indicates samples that tested positive for MAP in the representative laboratory.

**Table 2:** The top five phyla in each group.

Group	Dominant Phylum	Relative Abundance (%)
	Samples in PTB Animal Model	
M1	p. Firmicutes	58.44
	p. Bacteroidota	37.88
	p. Verrucomicrobiota	3.19
	p. Spirochaetota	1.86
	p. Patescibacteria	0.56
M2	p. Firmicutes	64.85
	p. Bacteroidota	24.21
	p. Spirochaetota.	5.62
	p. Verrucomicrobiota	2.87
	p. Proteobacteria	1.09
M3	p. Firmicutes	48.32
	p. Bacteroidota	41.54
	p. Verrucomicrobiota	3.97
	p. Spirochaetota	2.00
	p. Actinobacteriota	1.68
M4	p. Firmicutes	63.32
	p. Bacteroidota	24.66
	p. Verrucomicrobiota	8.72
	p. Spirochaetota	1.39
	p. Proteobacteria	0.44
M5	p. Firmicutes	66.56
	p. Bacteroidota	23.90
	p. Verrucomicrobiota	4.31
	p. Spirochaetota	1.98
	p. Proteobacteria	0.56
M6	p. Firmicutes	46.53
	p. Bacteroidota	30.81
	p. Spirochaetota	8.21
	p. Verrucomicrobiota	6.26
	p. Proteobacteria	1.05%
	Samples exposed to MAP in the field	
A	p. Firmicutes	65.81
	p. Bacteroidota	20.73
	p. Verrucomicrobiota	4.52
	p. Spirochaetota	2.67
	p. Proteobacteria	2.46
B	p. Firmicutes	44.66
	p. Bacteroidota	32.82
	p. Proteobacteria	14.97
	p. Verrucomicrobiota	2.31
	p. Spirochaetota	2.21
C	p. Firmicutes	51.90
	p. Bacteroidota	24.10
	p. Proteobacteria	16.81
	p. Actinobacteriota	2.59
	p. Spirochaetota	2.57

**Table 3:** Top five genera in each group.

Group	Dominant Genus	Relative Abundance (%)
	Samples in PTB Animal Model	
M1	g. UCG-005	11.19
	g. Clostridia_UCG-014_norank	10.17
	g. Christensenellaceae_R-7_group	9.07
	g. Bacteroides	7.25
	g. Alistipes	5.63
M2	g. Christensenellaceae_R-7_group	7.49
	g. UCG-005	7.45
	g. Rikenellaceae_RC9_gut_group	7.18
	g. Streptococcus	6.04
	g. Treponema	5.57
M3	g. Rikenellaceae_RC9_gut_group	17.80
	g. Muribaculaceae_norank	5.66
	g. Alistipes	5.31
	g. UCG-005	5.00
	g. Bacteroides	4.95
M4	g. Akkermansia	8.30
	g. UCG-005	7.784
	g. Monoglobus	5.90
	g. Clostridia_UCG-014_norank	5.20
	g. Alistipes	5.10
M5	g. UCG-005	8.22
	g. Bacteroides	6.55
	g. [Eubacterium]_coprostanoligenes_group_norank	6.15
	g. Christensenellaceae_R-7_group	5.01
	g. UCG-010_norank	4.93
M6	g. Bacteroides	8.30
	g. Treponema	8.15
	g. Fibrobacter	5.99
	g. UCG-005	5.88
	g. Akkermansia	5.68
	Samples exposed to MAP in the field	
A	g. Christensenellaceae_R-7_group	10.09
	g. UCG-005	9.97
	g. Clostridia_UCG-014_norank	5.53
	g. Rikenellaceae_RC9_gut_group	5.30
	g. Lachnospiraceae_unclassified	4.33
B	g. Escherichia-Shigella	13.75
	g. Muribaculaceae_norank	9.43
	g. Bacteroides	8.23
	g. UCG-005	7.05
	g. Clostridia_UCG-014_norank	5.73
C	g. Acinetobacter	11.24
	g. Christensenellaceae_R-7_group	6.19
	g. UCG-005	5.64
	g. Rikenellaceae_RC9_gut_group	5.13
	g. Bacteroides	3.17

## Data Availability

The raw tags have been deposited in the Sequence Read Archive (SRA) from the NCBI under BioProject accession number PRJNA1158702. The individual run files received the accession numbers SAMN43545949~SAMN43546020 (Table S3).

## Supplementary Materials

The following supporting information can be downloaded at: https://www.mdpi.com/article/10.3390/pathogens13121118/s1, Table S1. Statistical analysis of the sequencing data of 72 samples. Table S2. Microbial richness and alpha diversity indices of the 72 samples. Table S3. Accession, sample name, SPUID, organism, TaxID, and BioProject.

## Abbreviations

PTB: Paratuberculosis; MAP: *Mycobacterium avium* subsp. *Paratuberculosis*; STHS: small-tail Han sheep; PCR: polymerase chain reaction; and OTUs: operational taxonomic units.
