# Supplementary material for: The Impact of Mycobacterium avium subsp. paratuberculosis on Intestinal Microbial Community Composition and Diversity in Small-Tail Han Sheep
Source: Pathogens. 2024 Dec 18;13(12):1118. doi: 10.3390/pathogens13121118 (PMC11680033; doi:10.3390/pathogens13121118)
Supplement: Supplementary file 1 [file pathogens-13-01118-s001.zip › pathogens-3350138-supplementary.pdf]

Table S1. Statistical analysis of the sequencing data of 72 samples.

Group	Sheep	Sequences (n)	Bases (bp)	Average Length (bp)
	Samples in PTB animal model			
M1	1-1	43005	17709323	411.8
	2-1	75019	30904849	411.96
	3-1	47847	19658013	410.85
	4-1	52056	21479151	412.62
	5-1	44091	18133882	411.28
	6-1	57982	23928085	412.68
	7-1	71617	29550106	412.61
	8-1	54367	22424313	412.46
M2	1-2	45476	18733299	411.94
	2-2	60130	24589384	408.94
	3-2	71784	29613976	412.54
	4-2	50344	20842121	413.99
	5-2	65939	27465453	416.53
	6-2	74016	30371946	410.34
	7-2	47778	19599941	410.23
	8-2	59156	24413024	412.69
M3	1-3	47470	19664147	414.24
	2-3	69278	28598851	412.81
	3-3	50688	20935590	413.03
	4-3	58125	24031749	413.45
	5-3	67919	28053371	413.04
	6-3	70682	29150471	412.42
	7-3	69387	28603722	412.23
	8-3	79557	32917209	413.76
M4	1-4	68185	27926548	409.57
	2-4	70638	29051016	411.27
	3-4	70077	28797980	410.95
	4-4	79460	32731392	411.92
	5-4	118760	48561708	408.91
	6-4	68930	28292627	410.45
	7-4	65069	26750784	411.11
	8-4	84366	34559544	409.64
M5	1-5	144951	59411061	409.87
	2-5	74296	30644964	412.47
	3-5	62357	25497452	408.89
	4-5	81331	33419534	410.91
	5-5	107734	44305461	411.25
	6-5	57751	23675406	409.96
	7-5	73455	30228488	411.52
	8-5	47108	19368950	411.16
M6	1-6	120499	49806785	413.34
	2-6	60382	25085610	415.45
	3-6	71565	29507147	412.31
	4-6	45552	18735589	411.3
	5-6	74806	31008887	414.52
	6-6	53459	22176365	414.83
	7-6	53872	22225686	412.56

	8-6	45371	18778659	413.89
	Samples exposed to MAP in the field			
Group A	A1	52128	21446652	411.42
	A2	56818	23452606	412.77
	A3	45160	18480084	409.21
	A4	48628	20140677	414.18
	A5	166011	68580841	413.11
	A6	168363	69719105	414.1
	A7	45982	19013466	413.5
	A8	31949	13149785	411.59
Group B	B1	59539	24818836	416.85
	B2	47685	20073864	420.97
	B3	46178	19206470	415.92
	B4	51388	21814321	424.5
	B5	41884	17286964	412.73
	B6	155097	64122994	413.44
	B7	161537	67035090	414.98
	B8	44434	18408419	414.29
Group C	C1	59868	24923057	416.3
	C2	68391	28524241	417.08
	C3	52262	21680108	414.84
	C4	62038	25741925	414.94
	C5	60280	25451235	422.22
	C6	393633	166841328	423.85
	C7	357305	148515425	415.65
	C8	72229	29999575	415.34

Table S2. Microbial richness and alpha diversity indices of the 72 samples.

Sample name	0.97						
	Reads	OTU	ACE index	Chao index	Coverage (%)	Shannon index	Simpson index
1-1	11569	644	762 (729, 806)	736 (705, 783)	0.986948	4.75 (4.72, 4.78)	0.0293 (0.0279, 0.0308)
1-2	13365	722	845 (812, 890)	820 (787, 868)	0.988178	5.15 (5.12, 5.17)	0.0153 (0.0146, 0.016)
1-3	17721	504	596 (568, 637)	589 (557, 639)	0.993398	4.13 (4.1, 4.16)	0.0441 (0.0427, 0.0454)
1-4	19543	922	1036 (1006, 1076)	990 (967, 1025)	0.991659	5.39 (5.36, 5.41)	0.0109 (0.0105, 0.0112)
1-5	61874	1292	1408 (1379, 1448)	1375 (1349, 1415)	0.997107	5.53 (5.51, 5.54)	0.0095 (0.0093, 0.0097)
1-6	49382	1210	1332 (1302, 1373)	1285 (1261, 1320)	0.996355	5.19 (5.18, 5.21)	0.0169 (0.0165, 0.0172)
2-1	27344	880	998 (967, 1040)	986 (952, 1037)	0.993746	5.03 (5, 5.05)	0.0172 (0.0167, 0.0177)
2-2	23064	751	875 (841, 921)	846 (814, 895)	0.993366	4.91 (4.89, 4.94)	0.0252 (0.0242, 0.0262)
2-3	23026	841	959 (928, 1000)	927 (898, 968)	0.992791	4.85 (4.83, 4.88)	0.0207 (0.0201, 0.0213)

2-4	25828	931	1066 (1032, 1112)	1029 (998, 1074)	0.992992	5.08 (5.06, 5.1)	0.0185 (0.018, 0.0191)
2-5	36525	1062	1163 (1135,1200)	1165 (1131, 1215)	0.995619	5.42 (5.4, 5.44)	0.0177 (0.017, 0.0184)
2-6	21247	809	912 (884, 950)	890 (862, 932)	0.992940	4.89 (4.86, 4.91)	0.023 (0.0223, 0.0238)
3-1	15826	639	773 (736, 823)	745 (710, 798)	0.990143	4.71 (4.68, 4.74)	0.0217 (0.021, 0.0224)
3-2	36096	758	889 (853, 938)	876 (837, 935)	0.995623	4.81 (4.79, 4.83)	0.019 (0.0186, 0.0194)
3-3	22413	634	707 (684, 741)	716 (685, 765)	0.994914	4.63 (4.61, 4.66)	0.0296 (0.0286, 0.0306)
3-4	19085	873	999 (967, 1042)	949 (924, 986)	0.991040	4.91 (4.88, 4.94)	0.0272 (0.0261, 0.0283)
3-5	27661	964	1103 (1068, 1149)	1082 (1045, 1135)	0.993348	5.32 (5.31, 5.34)	0.0124 (0.0121, 0.0127)
3-6	28597	988	1128 (1093, 1175)	1119 (1079, 1178)	0.993321	5.28 (5.26, 5.3)	0.0138 (0.0134, 0.0142)
4-1	16543	646	771 (737, 818)	730 (701, 774)	0.990993	4.47 (4.44, 4.5)	0.036 (0.0347, 0.0372)
4-2	26106	546	652 (620, 697)	657 (616, 721)	0.995212	3.93 (3.9, 3.95)	0.071 (0.0691, 0.0729)
4-3	27552	797	933 (898, 982)	917 (878, 974)	0.993902	4.69 (4.67, 4.71)	0.0291 (0.0282, 0.0299)
4-4	32776	947	1074 (1042, 1117)	1031 (1004, 1071)	0.994600	4.14 (4.11, 4.16)	0.1195 (0.1161, 0.1228)
4-5	24560	1093	1235 (1201, 1280)	1199 (1167, 1244)	0.991612	5.38 (5.36, 5.4)	0.0134 (0.0129, 0.0138)
4-6	15210	768	918 (880, 968)	906 (863, 968)	0.987771	4.93 (4.9, 4.96)	0.0205 (0.0197, 0.0213)
5-1	16342	683	830 (791, 884)	811 (769, 874)	0.990026	5.08 (5.05, 5.1)	0.0138 (0.0133, 0.0142)
5-2	42182	737	840 (811, 880)	850 (811, 909)	0.996515	3.22 (3.2, 3.25)	0.2475 (0.2428, 0.2521)
5-3	26702	674	762 (736, 799)	745 (719, 786)	0.995319	3.99 (3.96, 4.02)	0.0788 (0.0766, 0.081)
5-4	43831	1080	1194 (1165, 1233)	1160 (1134, 1199)	0.996076	5.2 (5.18, 5.21)	0.0144 (0.0141, 0.0147)
5-5	38438	1206	1347 (1313, 1391)	1301 (1272, 1342)	0.994667	5.42 (5.4,5.44)	0.012 (0.0117, 0.0123)
5-6	35296	895	996 (968, 1035)	990 (958, 1038)	0.995637	4.64 (4.62, 4.66)	0.0499 (0.0483, 0.0515)
6-1	19938	829	959 (925, 1005)	942 (906, 995)	0.991273	5.19 (5.17, 5.22)	0.0126 (0.0122, 0.013)
6-2	21763	922	1053 (1020, 1097)	1000 (975, 1038)	0.992005	5.21 (5.19, 5.23)	0.0148 (0.0143, 0.0153)
6-3	23825	880	991 (962, 1031)	949 (925, 985)	0.993494	4.71 (4.68, 4.74)	0.0579 (0.0556, 0.0603)
6-4	29076	934	1098 (1058, 1152)	1081 (1036, 1144)	0.993190	5.21 (5.2, 5.23)	0.0144 (0.014, 0.0148)
6-5	17488	970	1116 (1080, 1162)	1071 (1040, 1116)	0.988907	5.53 (5.51, 5.56)	0.0088 (0.0085, 0.0091)
6-6	23359	781	936 (897, 989)	929 (883, 997)	0.992209	4.27 (4.24, 4.3)	0.0991 (0.0956, 0.1026)
7-1	38516	873	1009 (973, 1058)	1019 (972, 1088)	0.995534	5.11 (5.1, 5.13)	0.013 (0.0127, 0.0132)
7-2	17970	775	924 (886, 976)	906 (864, 966)	0.990039	5.14 (5.12, 5.16)	0.0134 (0.013, 0.0138)
7-3	25813	724	818 (792, 855)	783 (762, 816)	0.994731	3.9 (3.87, 3.93)	0.1176 (0.1139, 0.1212)

7-4	26990	918	1046 (1013, 1092)	1032 (995, 1085)	0.993590	5.28 (5.26, 5.3)	0.0121 (0.0118, 0.0123)
7-5	28705	1033	1180 (1144, 1228)	1188 (1141, 1256)	0.993033	5.34 (5.32, 5.36)	0.0144 (0.014, 0.0149)
7-6	15412	815	943 (910, 987)	894 (868, 933)	0.988905	4.98 (4.95, 5.01)	0.0265 (0.0252, 0.0277)
8-1	25595	692	825 (789, 875)	807 (769, 864)	0.993827	4.47 (4.45, 4.5)	0.0332 (0.0323, 0.034)
8-2	24035	710	869 (828, 925)	864 (815, 936)	0.992677	4.81 (4.79, 4.83)	0.0205 (0.0199, 0.0211)
8-3	30086	586	656 (634, 688)	627 (611, 655)	0.996576	3.84 (3.82, 3.86)	0.0857 (0.0832, 0.0882)
8-4	24459	957	1077 (1046, 1118)	1028 (1004, 1062)	0.993009	5.25 (5.22, 5.27)	0.0127 (0.0123, 0.013)
8-5	14236	850	968 (937, 1009)	950 (918, 999)	0.988269	5.47 (5.45, 5.5)	0.0105 (0.0101, 0.011)
8-6	14185	682	781 (753, 821)	758 (731, 798)	0.990201	4.82 (4.79, 4.85)	0.0252 (0.024, 0.0264)
A1	49226	927	1016 (991, 1051)	996 (971, 1034)	0.997258	5.09 (5.08, 5.11)	0.0132 (0.0129, 0.0134)
A2	53267	1582	1757 (1718, 1807)	1754 (1706, 1819)	0.995325	5.99 (5.98, 6.01)	0.0073 (0.0071, 0.0075)
A3	42488	967	1091 (1058, 1135)	1069 (1036, 1119)	0.996093	5.31 (5.3, 5.33)	0.0103 (0.0101, 0.0105)
A4	45714	1548	1743 (1170, 1796)	1774 (1714, 1855)	0.993984	5.9 (5.89, 5.92)	0.0072 (0.007, 0.0073)
A5	160189	2379	2379 (2379, 2379)	2379 (2379, 2379)	1.000000	5.92 (5.92, 5.93)	0.0069 (0.0068, 0.007)
A6	161413	2576	2576 (2576, 2576)	2576 (2576, 2576)	1.000000	6.12 (6.11, 6.13)	0.0052 (0.0052, 0.0053)
A7	43505	1484	1662 (1623, 1713)	1664 (1614, 1732)	0.994208	5.97 (5.96, 5.99)	0.0062 (0.0061, 0.0064)
A8	29941	1342	1513 (1474, 1563)	1555 (1496, 1636)	0.991717	6.05 (6.03, 6.06)	0.0052 (0.0051, 0.0054)
B1	57268	445	532 (503, 577)	551 (508, 625)	0.998341	3.94 (3.93, 3.96)	0.0437 (0.043, 0.0444)
B2	45760	757	874 (841, 918)	881 (839, 944)	0.996656	3.15 (3.12, 3.17)	0.2768 (0.2721, 0.2815)
B3	44504	385	412 (400, 433)	405 (394, 427)	0.998966	4.07 (4.05, 4.08)	0.0315 (0.031, 0.0319)
B4	50033	256	300 (282, 331)	299 (278, 339)	0.998881	1.73 (1.71, 1.74)	0.338 (0.3352, 0.3407)
B5	39587	1357	1494 (1461, 1538)	1510 (1465, 1574)	0.994594	5.96 (5.94, 5.97)	0.0059 (0.0058, 0.0061)
B6	149529	2365	2365 (2365, 2365)	2365 (2365, 2365)	1.000000	6.12 (6.11, 6.13)	0.005 (0.0049, 0.0051)
B7	155751	2297	229 (2297, 2297)	2297 (2297, 2297)	1.000000	6.05 (6.04, 6.06)	0.0057 (0.0056, 0.0057)
B8	41930	1412	1591 (1551, 1643)	1593 (1542, 1662)	0.994181	5.93 (5.91, 5.94)	0.0062 (0.0006, 0.0063)
C1	56220	1861	2136 (2084, 2202)	2142 (2075, 2229)	0.993668	6.08 (6.07, 6.09)	0.0055 (0.0054, 0.0056)
C2	64126	1860	2148 (2093, 2215)	2166 (2094, 2260)	0.994308	5.87 (5.86, 5.88)	0.0102 (0.0099, 0.0105)
C3	48876	1825	2092 (2041, 2155)	2131 (2058, 2227)	0.992757	6.09 (6.08, 6.11)	0.0058 (0.0057, 0.0059)
C4	58430	1412	1614 (1570, 1671)	1635 (1574, 1717)	0.995482	5.52 (5.51, 5.54)	0.0132 (0.0129, 0.0135)

C5	56330	1573	1893 (1833, 1966)	1900 (1825, 1999)	0.993254	4.51 (4.49, 4.53)	0.0655 (0.0642, 0.0668)
C6	375707	5661	5661 (5661, 5661)	5661 (5661, 5661)	1.000000	4.7 (4.7, 4.71)	0.0417 (0.0414, 0.042)
C7	337581	6029	6029 (6029, 6029)	6029 (6029, 6029)	1.000000	6.08 (6.07, 6.09)	0.0117 (0.0115, 0.0118)
C8	68235	1828	2065 (2018, 2125)	2117 (2046, 2213)	0.995222	5.98 (5.97, 5.99)	0.0076 (0.0074, 0.0078)

Note: In (X, Y) values, X represents the statistically significant lower limit value and Y represents the statistically significant upper limit value.

Table S3. Sample name, accession number, and BioProject ID.

Sample name	Accession number	BioProject ID
1-1	SAMN43545949	PRJNA1158702
1-2	SAMN43545950	PRJNA1158702
1-3	SAMN43545951	PRJNA1158702
1-4	SAMN43545952	PRJNA1158702
1-5	SAMN43545953	PRJNA1158702
1-6	SAMN43545954	PRJNA1158702
2-1	SAMN43545955	PRJNA1158702
2-2	SAMN43545956	PRJNA1158702
2-3	SAMN43545957	PRJNA1158702
2-4	SAMN43545958	PRJNA1158702
2-5	SAMN43545959	PRJNA1158702
2-6	SAMN43545960	PRJNA1158702
3-1	SAMN43545961	PRJNA1158702
3-2	SAMN43545962	PRJNA1158702
3-3	SAMN43545963	PRJNA1158702
3-4	SAMN43545964	PRJNA1158702
3-5	SAMN43545965	PRJNA1158702
3-6	SAMN43545966	PRJNA1158702
4-1	SAMN43545967	PRJNA1158702
4-2	SAMN43545968	PRJNA1158702
4-3	SAMN43545969	PRJNA1158702
4-4	SAMN43545970	PRJNA1158702
4-5	SAMN43545971	PRJNA1158702
4-6	SAMN43545972	PRJNA1158702
5-1	SAMN43545973	PRJNA1158702
5-2	SAMN43545974	PRJNA1158702
5-3	SAMN43545975	PRJNA1158702
5-4	SAMN43545976	PRJNA1158702
5-5	SAMN43545977	PRJNA1158702
5-6	SAMN43545978	PRJNA1158702
6-1	SAMN43545979	PRJNA1158702
6-2	SAMN43545980	PRJNA1158702
6-3	SAMN43545981	PRJNA1158702
6-4	SAMN43545982	PRJNA1158702
6-5	SAMN43545983	PRJNA1158702
6-6	SAMN43545984	PRJNA1158702
7-1	SAMN43545985	PRJNA1158702

7-2	SAMN43545986	PRJNA1158702
7-3	SAMN43545987	PRJNA1158702
7-4	SAMN43545988	PRJNA1158702
7-5	SAMN43545989	PRJNA1158702
7-6	SAMN43545990	PRJNA1158702
8-1	SAMN43545991	PRJNA1158702
8-2	SAMN43545992	PRJNA1158702
8-3	SAMN43545993	PRJNA1158702
8-4	SAMN43545994	PRJNA1158702
8-5	SAMN43545995	PRJNA1158702
8-6	SAMN43545996	PRJNA1158702
A1	SAMN43545997	PRJNA1158702
A2	SAMN43545998	PRJNA1158702
A3	SAMN43545999	PRJNA1158702
A4	SAMN43546000	PRJNA1158702
A5	SAMN43546001	PRJNA1158702
A6	SAMN43546002	PRJNA1158702
A7	SAMN43546003	PRJNA1158702
A8	SAMN43546004	PRJNA1158702
B1	SAMN43546005	PRJNA1158702
B2	SAMN43546006	PRJNA1158702
B3	SAMN43546007	PRJNA1158702
B4	SAMN43546008	PRJNA1158702
B5	SAMN43546009	PRJNA1158702
B6	SAMN43546010	PRJNA1158702
B7	SAMN43546011	PRJNA1158702
B8	SAMN43546012	PRJNA1158702
C1	SAMN43546013	PRJNA1158702
C2	SAMN43546014	PRJNA1158702
C3	SAMN43546015	PRJNA1158702
C4	SAMN43546016	PRJNA1158702
C5	SAMN43546017	PRJNA1158702
C6	SAMN43546018	PRJNA1158702
C7	SAMN43546019	PRJNA1158702
C8	SAMN43546020	PRJNA1158702
